# The Loss of the Inverted Repeat in the Putranjivoid Clade of Malpighiales

**DOI:** 10.3389/fpls.2020.00942

**Published:** 2020-06-26

**Authors:** Dong-Min Jin, Susann Wicke, Lu Gan, Jun-Bo Yang, Jian-Jun Jin, Ting-Shuang Yi

**Affiliations:** ^1^ Germplasm Bank of Wild Species, Kunming Institute of Botany, Chinese Academy of Sciences, Kunming, China; ^2^ College of Life Sciences, University of Chinese Academy of Sciences, Beijing, China; ^3^ Institute for Evolution and Biodiversity, University of Münster, Münster, Germany

**Keywords:** plastome evolution, Inverted Repeat region loss, genomic rearrangement, Lophopyxidaceae, Putranjivaceae

## Abstract

The typical plastid genome (plastome) of photosynthetic angiosperms comprises a pair of Inverted Repeat regions (IRs), which separate a Large Single Copy region (LSC) from a Small Single Copy region (SSC). The independent losses of IRs have been documented in only a few distinct plant lineages. The majority of these taxa show uncommonly high levels of plastome structural variations, while a few have otherwise conserved plastomes. For a better understanding of the function of IRs in stabilizing plastome structure, more taxa that have lost IRs need to be investigated. We analyzed the plastomes of eight species from two genera of the putranjivoid clade of Malpighiales using Illumina paired-end sequencing, the *de novo* assembly strategy GetOrganelle, as well as a combination of two annotation methods. We found that all eight plastomes of the putranjivoid clade have lost their IR_B_, representing the fifth case of IR loss within autotrophic angiosperms. Coinciding with the loss of the IR, plastomes of the putranjivoid clade have experienced significant structural variations including gene and intron losses, multiple large inversions, as well as the translocation and duplication of plastome segments. However, Balanopaceae, one of the close relatives of the putranjivoid clade, exhibit a relatively conserved plastome organization with canonical IRs. Our results corroborate earlier reports that the IR loss and additional structural reorganizations are closely linked, hinting at a shared mechanism that underpins structural disturbances.

## Introduction

Plastids, such as chloroplasts, chromoplasts, and leucoplasts, are the place for photosynthesis and the major organelle for organic product storage in plants. Plastids retain a semi-autonomous genetic system with their own genome (plastome). Typically, the plastome of a photosynthetic angiosperm is a circular molecule, with a length of 120–160 kb (Wicke et al., 2011). Structurally, such plastome comprises a pair of Inverted Repeat regions (hereafter called IRs; ~25 kb), a Large Single Copy region (LSC; ~85 kb), and a Small Single Copy region (SSC; ~15 kb) (Ruhlman and Jansen, 2014; Mower and Vickrey, 2018). IRs may play an important role in maintaining plastome stability (Maréchal and Brisson, 2010), which might be one of the reasons why most autotrophic angiosperms possess canonical IRs. However, IR losses have been documented in a few distinct angiosperm lineages, namely the IR-Lacking Clade (IRLC) of Leguminosae (Palmer and Thompson, 1981; Palmer and Thompson, 1982; but see Choi et al., 2019), two *Erodium* lineages of Geraniaceae (Guisinger et al., 2011; Ruhlman et al., 2017), *Carnegiea gigantea* of Cactaceae, and *Tahina spectabilis* of Arecaceae (Choi et al., 2019). Plastomes of the IRLC, *C. gigantea* (Sanderson et al., 2015), *Tahina spectabilis* (Barrett et al., 2016) showed significant higher rearrangement degrees compared to their sister clade, while species in a lineage of *Erodium* that has lost one IR exhibit an otherwise conserved plastome structure (Blazier et al., 2016). Hence, further comparative study is needed to elucidate the function of IRs in stabilizing plastome structure.

Malpighiales are one of the largest orders of flowering plants. Plants in this order exhibit a remarkable morphological and ecological diversity, with many species of great ecological and economic importance (Xi et al., 2012). Previous studies have revealed significant structural variations in the plastomes of multiple taxa in this order. Rabah et al. (2019) compared plastomes of 15 species of the genus *Passiflora* (Passifloraceae) and found that this genus has experienced widespread genomic changes, including inversions, gene and intron losses along with multiple independent IR expansions and contractions. Lopes et al. (2018) revealed the contraction and expansion of the IRs altering the size, gene content, and gene order of SC and IRs in the plastome of *Linum usitatissimum* (Linaceae). Tangphatsornruang et al. (2011) reported a 30-kb inversion between *trnE*-UUC—*trnS*-GCU and *trnT*-GGU—*trnR*-UCU in *Hevea brasiliensis* (Euphorbiaceae). Two recent studies detected an inversion in the LSC, significant variation in length reduction of the IRs, gene loss and pseudogenization events in plastomes of Podostemaceae (Bedoya et al., 2019; Jin et al., 2020). An inversion over 50 kb spanning from *trnK*-*UUU* to *rbcL* in the LSC is shared by *Cratoxylum cochinchinense* (Hypericaceae), *Tristicha trifaria,* and *Marathrum* *foeniculaceum* (Podostemaceae) (Jin et al., 2020). Previous studies suggested that multiple lineages of Malpighiales have experienced plastome structural variations, but knowledge of plastomes evolution in this large order is still limited.

The putranjivoid clade in Malpighiales consists of two families: Lophopyxidaceae and Putranjivaceae (Wurdack and Davis, 2009). Lophopyxidaceae have a single genus, whereas Putranjivaceae contain three genera and ca. 216 species. Containing 209 species, *Drypetes* is the largest genus in Putranjivaceae. The species in this clade are perennial trees or shrubs, growing primarily in tropical and subtropical areas (Kubitzki, 2014).

As it is unknown to date how plastid genomes evolve in the putranjivoid clade, we here assembled the complete plastome sequences for eight species, as well as two species from the closely related family Balanopaceae, representing one genus each from each family. Our analyses focused on exploring the structural variation of plastomes and revealed that all plastomes of the putranjivoid calde have lost the IR_B_ entirely and experienced extensive additional structural rearrangements. In contrast, the plastomes of the two Balanopaceae species retain a relatively conserved plastome structure, indicating an evolutionary shift after the split of both lineages.

## Materials and Methods

### Taxon Sampling, DNA Extraction and Sequencing

We sampled seven species from the largest genus *Drypetes* of Putranjivaceae, one species from Lophopyxidaceae, and two species from Balanopaceae as outgroups. Total genomic DNA of all samples was isolated from herbarium specimens or silica gel-dried leaves using the DNeasy Plant Mini Kit (Tiangen Biotech Co., LTD., Beijing, China) or a standardized CTAB-protocol (Doyle and Doyle, 1987). Following quantity checks and library preparations, paired-end sequencing was carried out on Illumina HiSeq 2000 or HiSeq X TEN at the Plant Germplasm and Genomics Center (Kunming Institute of Botany, Chinese Academy of Sciences). A genome skimming sequencing approach was employed. **Table S1** provides original collection location, herbarium voucher information, GenBank accession numbers, as well as the read characteristics for all taxa discussed in this study.

### Plastome Assembly and Annotation

Plastomes were assembled using GetOrganelle v1.6.1a with default settings, which filtered plastid-like reads, conducted the *de novo* assembly, purified the assembly graph, and generated the complete plastomes (Camacho et al., 2009; Bankevich et al., 2012; Langmead and Salzberg, 2012; Jin et al., 2019). *K*-mer gradients were set according to the sequenced read lengths as “-k 21,31,41,51,65,85,91,95,99,101,111,121,127” for 150 bp reads; “-k 21,31,41,51,61,71,81,85,87” were used for 90 bp reads. Final assembly graphs were visualized in Bandage (Wick et al., 2015) to confirm the automatically generated plastomes. Two configurations of each plastome caused by the flip-flop recombination mediated by the IR or the ~1.2 kb sIR (short Inverted Repeat regions) were obtained, and one of them was arbitrarily selected for downstream analysis (Walker et al., 2015). All plastomes were initially annotated using PGA (Qu et al., 2019) and GeSeq (Tillich et al., 2017), with the annotated plastome of *Amborella trichopoda* (NC_005086) (Goremykin et al., 2003) selected as the reference. For confirmation, all annotations were compared with the previously published plastome of *Byrsonima coccolobifolia* (NC_037191; Malpighiaceae; Menezes et al., 2018) and manually examined in Geneious Prime (https://www.geneious.com). All newly sequenced plastomes were deposited in GenBank under accession numbers MN504788–MN504797.

### Phylogenetic Analysis

Phylogenetic analysis was performed using 71 protein-coding genes, which were shared by all study species (**Table S2**). Gene sequences were extracted using get_annotated_regions_from_gb.py (https://github.com/Kinggerm/PersonalUtilities, accessed on July 30, 2019; Zhang et al., 2020), aligned individually using prank v.140603 (Loytynoja and Goldman, 2008), then concatenated into a single aligned dataset using concatenate_fasta.py (https://github.com/Kinggerm/PersonalUtilities, accessed on July 30, 2019; Zhang et al., 2020). To reconstruct the phylogenetic relationships among our taxa, we employed RAxML v.8.2.11 (Stamatakis, 2014) with “-m GTRGAMMA”, which performs tree searches and optimization under the maximum likelihood paradigm. For statistical support, we ran 1,000 bootstrap replicates, and visualized the results in FigTree v.1.4.4 (http://tree.bio.ed.ac.uk). We mapped the events manually, facilitated by the small size of the data set, assuming that pseudogenization, gene loss, and IR loss are irreversible events.

### Plastome Structural Rearrangements

To build whole plastome alignments for the putranjivoid clade, and the two *Balanops* species, we used the progressiveMauve algorithm in Mauve v2.3.1 (Darling et al., 2010) with default settings. The IR_B_ was removed from plastid genomes with two copies of the large inverted repeats to allow for an optimal homology assessment (Wicke et al., 2013). Based on the strand orientation of the Locally Collinear Blocks (LCBs) identified by the progressiveMauve alignment, strand orientation determines the sign (+/-). Compared with the references, each LCB was numbered. Subsequently, we used GRIMM (Tesler, 2002) to calculate genome rearrangement distances.

### Number of Repeats

Dispersed repeats (including forward, reverse, complement, and palindromic repeats) were identified by REPuter (Kurtz et al., 2001) based on the following criteria: minimum repeat size ≥ 30 bp; sequence identities ≥ 90%; Hamming distance = 3. Again, the IR_B_ was removed, where present. REPuter overestimates the number of repetitive elements in a given sequence by recognizing nested or overlapping repeats within a given region containing multiple repeats (Wang et al., 2018). The FindRepeats plugin of Geneious Prime was also used to identify repeated regions using a minimum repeat length of 30 bp and zero mismatches.

### Confirmation of 271 bp sIR-Induced Isomers

sIR range from 11 bp to several kbs in plastomes and are capable of inducing plastomic inversions and isomer (Martin et al., 2014; Wang et al., 2018). As sIR can potentially induce isomers, we used the library information of paired-end reads to confirm the existence of each potential isomers in *Lophopyxis maingayi*. We mapped the paired-end reads to the plastome sequence of each isomer, visually inspected the mapped read pairs in Geneious, and verified the existence of properly-mapped read pairs spanning the entire sIR. An isomer with read pairs spanning the entire sIR was supported to exist. Specifically, we firstly conducted read mapping using the evaluate_assembly_using_mapping.py script from the GetOrganelle toolkit, which calls Bowtie2 (Langmead and Salzberg, 2012). Because of the relatively short average insert size (**Table S1**), most read pairs are too short in insert size for providing confirmation and hampered visual inspection. For better visualization, we filtered the alignment using SAMtools (Li et al., 2009) by keeping records with an insert size between 330 and 600. Finally, we imported the filtered alignment file (*.sam) into Geneious Prime, turn on the “Layout-Link paired reads” mode and checked whether there are read pairs spanning the entire sIR.

## Results and Discussion

**Figure 1:**
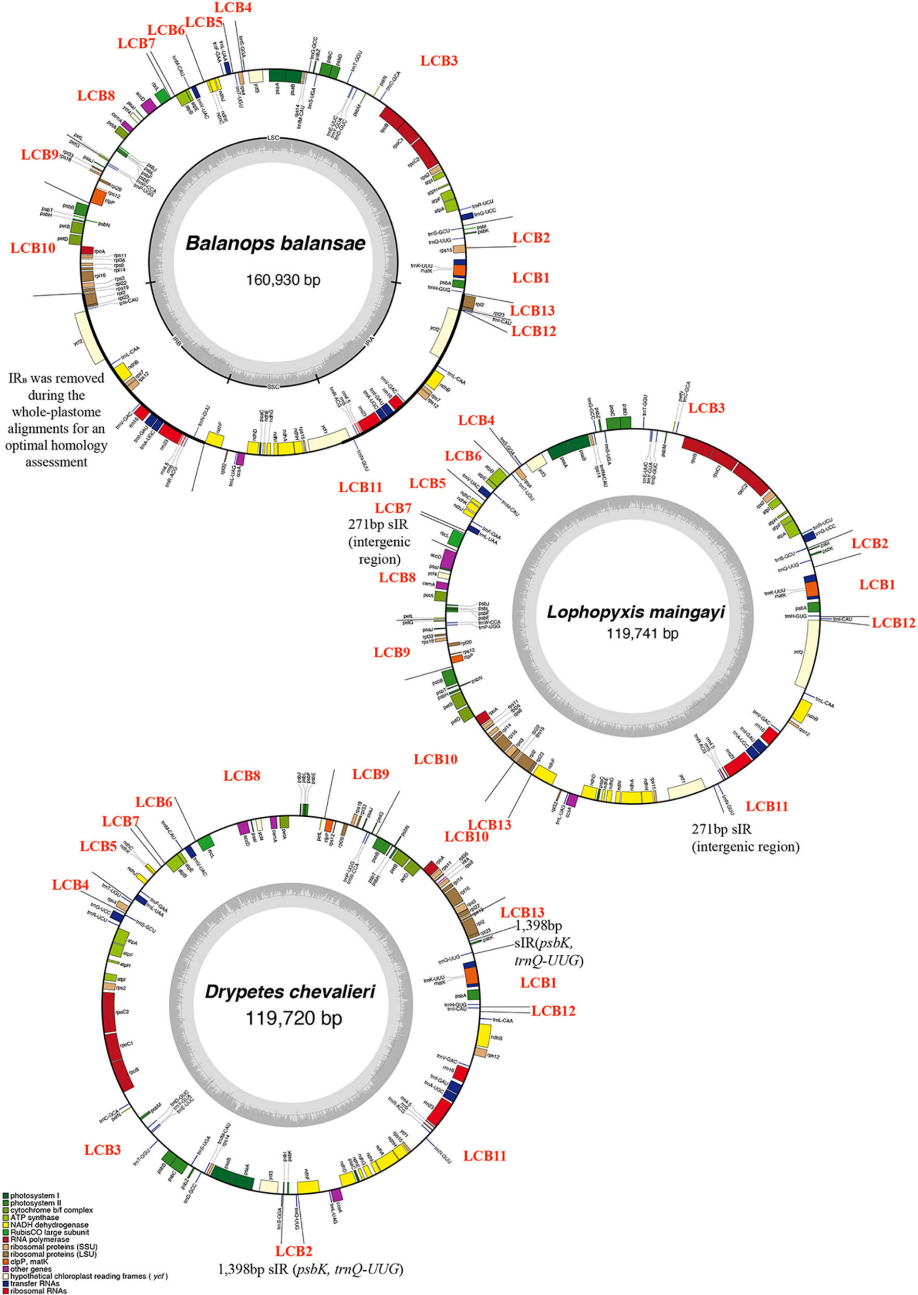
Plastid genomes of three species of Malpighiales representing three genera involved in this study. GC content graphs are shown as dark gray bars toward the center of each diagram. Each Locally Collinear Block (LCB) was indicated in the circular map, as well as the ~12kb short Inverted Repeat (sIR) and 271bp sIR.

Due to the differences in plant materials, the average base coverages of plastomes varied from 72 x to 640 x (**Table S1**). However, all ten newly assembled plastomes were complete. Plastomes from the putranjivoid clade are relatively small compared to their sister family Balanopaceae (**Figure 1**; **Table 1**). Variation in plastome size of the sampled putranjivoid sepcecies was small: *Drypetes hainanensis* has the smallest plastome with a length of 119,105 bp, while *Drypetes lateriflora* has the largest plastome with a length of 120,800 bp.

**Table 1:** Plastome features of 10 Malpighiales species.

Species	Plastome size (bp)	IR size (bp)	Number of unique genes*	sIR (bp)	Estimated rearrangement distance†
*Drypetes chevalieri*	119,720	n.a.	106	1,398	7
*Drypetes diopa*	119,299	n.a.	106	1,357	7
*Drypetes hainanensis*	119,105	n.a.	106	1,191	7
*Drypetes indica*	120,596	n.a.	106	1,047	7
*Drypetes lateriflora*	120,800	n.a.	107	1,484	7
*Drypetes longifolia*	119,268	n.a.	106	1,260	7
*Drypetes similis*	119,507	n.a.	106	1,221	7
*Lophopyxis maingayi*	119,741	n.a.	109	271	3
*Balanops balansae*	160,930	26,748	112	np	–
*Balanops pedicellata*	160,765	26,738	112	np	–

*Number of unique genes refers to unique gene number within the whole plastome.^†^Gene order changes were calculated relative to references (Balanops).bp, basepair; IR, inverted repeat; n.a., not applicable due to no IR pair; sIR, short inverted repeat that might have induced isomers; np, not present.

**Figure 2:**
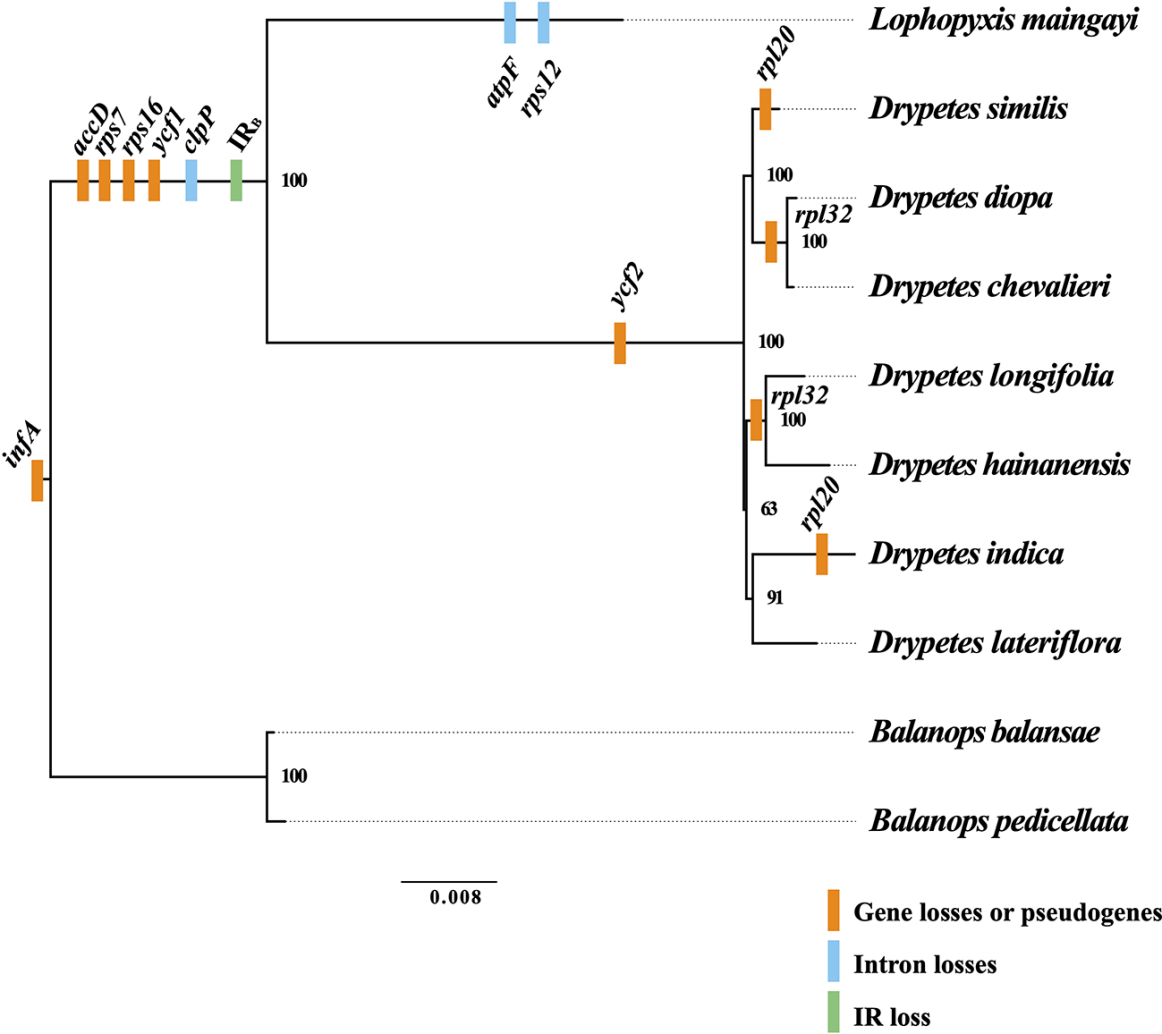
Gene losses or pseudogenes in the plastomes of the putranjivoid clade. Based on the phylogenetic tree, we mapped the events of gene and intron losses, as well as the event of the inverted repeat (IR) loss. Branch lengths correspond to substitutions per site.

Across autotrophic flowering plants, the content of IRs nearly universally includes all 4 rRNA genes, 7 tRNA genes, and a small number of protein genes (Mower and Vickrey, 2018). Plastomes of all studied putranjivoid species have lost a copy of the inverted repeat, namely IR_B_ (**Figure 1**, **Figure 2**; **Table 1**), which led to the observed significant reduction of their overall plastome size. All sampled putranjivoid species have lost the same segment of IR_B_ including 4 rRNA genes, 7 tRNA genes, and several protein coding genes (*rps12*, *rps7*, *ndhB*, *ycf2*, *rpl23*, and *rpl2*). Their plastome sizes were slightly varied due to the differences in intergenic regions. However, not all inversions are shared by *L. maingayi* and *Drypetes* species (**Figure 1**, **Table 2**).

**Table 2:** Permutations and Locally Collinear Blocks (LCB).

Species	Gene order
*Balanops balansae*	1, 2, 3, 4, 5, 6, 7, 8, 9, 10, 11, 12, 13
*Balanops pedicellata*	1, 2, 3, 4, 5, 6, 7, 8, 9, 10, 11, 12, 13
*Lophopyxis maingayi*	1, 2, 3, 4, -6, -5, 7, 8, 9, 10, -13, 11, 12
*Drypetes chevalieri*	1, 13, -10, 9, -8, 6, 7, -5, -4, 3, -2, 11, -12
*Drypetes diopa*	1, 13, -10, 9, -8, 6, 7, -5, -4, 3, -2, 11, -12
*Drypetes hainanensis*	1, 13, -10, 9, -8, 6, 7, -5, -4, 3, -2, 11, -12
*Drypetes indica*	1, 13, -10, 9, -8, 6, 7, -5, -4, 3, -2, 11, -12
*Drypetes lateriflora*	1, 13, -10, 9, -8, 6, 7, -5, -4, 3, -2, 11, -12
*Drypetes longifolia*	1, 13, -10, 9, -8, 6, 7, -5, -4, 3, -2, 11, -12
*Drypetes similis*	1, 13, -10, 9, -8, 6, 7, -5, -4, 3, -2, 11, -12

Negative numbers indicate a change of strand orientation.

To our knowledge, the IR loss event in the putranjivoid clade represents the fifth reported IR loss of autotrophic flowering plants. Among the five IR losses, the putranjivoid clade and *Tahina spectabilis* have lost IR_B_ (Barrett et al., 2016), while the IR-lacking legumes (Palmer and Thompson, 1981; Palmer and Thompson, 1982), *C. gigantea* (Sanderson et al., 2015), and some *Erodium* species (Guisinger et al., 2011; Ruhlman et al., 2017) all have lost their IR_A_. Which copy of IR has been lost seems to be a stochastic phenomenon. The two identical copies of the IR contain the same genes. None of the IR-lacking lineages, including all putranjivoid species, exhibits an impaired phenotype or habits (Blazier et al., 2016). Therefore, we may conclude that for those lineages one copy per IR-gene seems to be sufficient to support the overall function of the plastid.

The plastomes of Balanopaceae, one of the closest relatives of the putranjivoid clade, possess a canonical IR structure and a relatively conserved gene content and organization, which resembles those of the supposed ancestral angiosperm plastome (Ruhlman and Jansen, 2014). However, the plastomes of the putranjivoid clade have experienced significant gene content changes (**Table 1**; **Figure 2**). All examined plastomes from the putranjivoid clade lack intact *accD, rps7*, *rps16*, and *ycf1* genes (**Figure 2**), and all examined Putranjivaceae plastomes have one copy of the *ycf2* gene lost or became a pseudogene. The *rpl20* gene was inferred to be a pseudogene due to the presence of internal stop codons in the plastomes of *D. similis* and *D. indica* (**Figure S1**). *Drypetes diopa*, *D. chevalieri*, and *D. longifolia* lost the *rpl32* gene independently, and the *rpl32* gene of *D. hainanensis* was a pseudogene due to internal stop codons (**Figure S2**). The loss of *rps16* is common in angiosperm plastomes (Jansen et al., 2007). A study in *Medicago truncatula* (Leguminosae) and *Populus alba* (Salicaceae) showed that the *rps16* gene was lost in both species. However, the function of the plastid *rps16* was compensated by a nuclear-encoded *rps16* in both species (Ueda et al., 2008). The loss of *accD* in *Trifolium* species has been achieved by relocation to the nucleus (Magee et al., 2010). Two previous studies (Bedoya et al., 2019; Jin et al., 2020) suggested the uncommon loss or pseudogenization of *ycf1* and *ycf2* in Podostemaceae. Our results also suggested the loss or pseudogenization of *ycf1* in the putranjivoid clade, and the loss or pseudogenization of *ycf2* in Putranjivaceae. Moreover, all putranjivoid species lack both *clpP* introns, and *L. maingayi* lacks the typical introns in *atpF* and *rps12* (**Figure 2**). Previous studies indicated the loss of *rps12* and *clpP* introns in various legume lineages (Jansen et al., 2008; Wang et al., 2018). Recent studies on Podostemaceae also found the loss of both introns of *clpP* in riverweeds (Bedoya et al., 2019; Jin et al., 2020). The loss of the *atpF* intron was found not only in *Lophopyxis maingayi*, but also in members of Euphorbiaceae, Phyllanthaceae, Elatinaceae, and Passifloraceae of Malpighiales (Daniell et al., 2008). However, the mechanisms responsible for the intron losses remain elusive.

**Figure 3:**
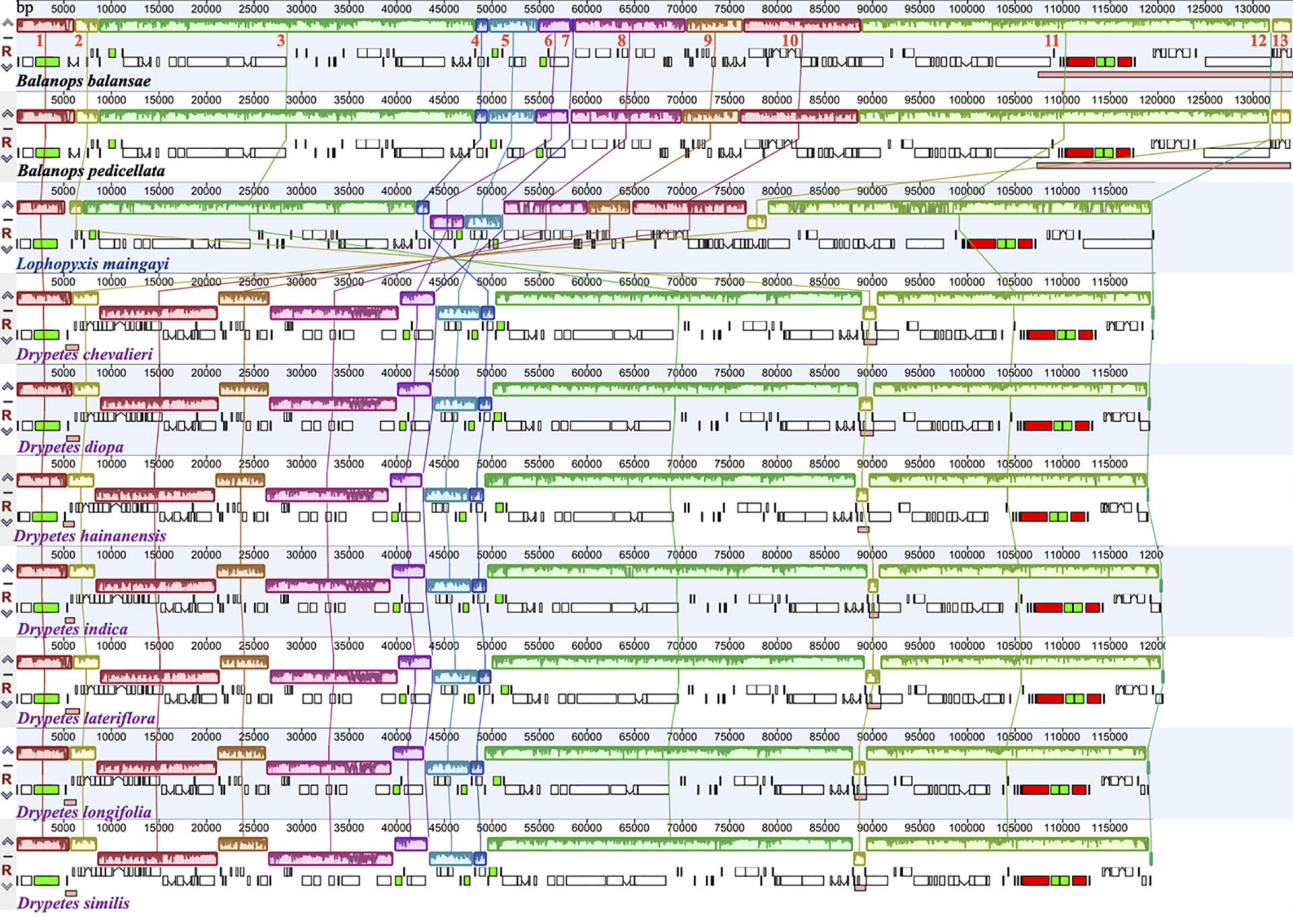
Plastid genome variation in the putranjivoid clade. Whole-plastome alignments divide the plastid genome of our study taxa into 13 Locally Collinear Blocks (LCB), which are shown as color-coded representations of syntenic regions. The IR_B_ was removed from plastid genomes with two copies of the large inverted repeats to allow for an optimal homology assessment. Blocks below the horizontal central line represent inversions relative to the references, shown as the upper two taxa. The height of the colored region within a block reflects the average sequence identity relative to the reference. Species names are color-coded to indicate their family: Balanopaceae (black), Lophopyxidaceae (blue), and Putranjivaceae (purple). The pink blocks in both *Balanops* species indicate the IR regions. Red blocks represent *rrn5*, *rrn4.5*, *rrn23*, and *rrn16* genes, green blocks represent *trnA*-UGC and *trnI*-GAU genes.

Plastomes of the putranjivoid clade have experienced notable structural reorganizations. Our progressiveMauve plastomes alignment of the putranjivoid clade with *Balanops* as references identified 13 syntenic regions (**Figures 1** and **3**, **Figures S3** and **S4**; **Table 2**). Genes or intergenic regions located in each LCB were identified (**Table 3**). Plastomic rearrangement distances were estimated based on the LCB orientations. The plastome of *L. maingayi* showed fewer rearrangements than those of Putranjivaceae species (**Figure 3**), as reflected in a lower genome rearrangement distance of 3 for *L. maingayi* but a higher genome rearrangement distance of 7 for the *Drypetes* species (**Table 1**). In *L. maingayi*, an inversion altered the syntenic blocks (4) (5) (6) (7) into (4) (-6) (-5) (7). LCB (5) and (6) corresponded to a 7.5-kb region between *atpB* and *trnL-UAA*. The order of the LCBs (10) (-13), (-13) (11), and the disruption of the adjacency of blocks (12) (13) were also the results of a translocation of LCB (13). LCB (13) corresponds to a 2-kb region spanning from the *rpl23* to the *rpl2* gene. Alternatively, a reasonable explanation for the changes around LCB (13) is that the *rpl23* and *rpl2* genes located in IR_A_ were lost, while the identical though inverted copies of these two genes from IR_B_ remained intact. Plastomes of all *Drypetes* species shared all inversions (**Figure 3**, **Figure S3**, and **Figure S4**). One optimal reversal (means rearrangement event such as inversion) scenario included 7 inversion events, which means the minimum number of inversions required for transforming in gene order from a *Drypetes* plastome to a *Balanops* plastome is 7.

**Table 3:** Genes in Locally Collinear Blocks (LCB) identified using ProgressiveMauve alignment for plastomes of the putranjivoid clade.

LCB	Genes
1	*trnH-GUG, psbA, trnK-UUU, matK*
2	*psbI, psbK, trnQ-UUG*
3	*trnS-GCU, trnG-UCC, trnR-UCU, atpA, atpF, atpH, atpI, rps2, rpoC2, rpoC1, rpoB, trnC-GCA, petN, psbM, trnD-GUC, trnY-GUA, trnE-UUC, trnT-GGU, psbD, psbC, trnS-UGA, psbZ, trnG-UCC, trnM-CAU, rps14, psaB, psaA, ycf3, trnS-GGA*
4	*trnT-UGU, rps4*
5	*ndhC, ndhK, ndhJ, trnF-GAA, trnL-UAA*
6	*trnV-UAC, trnM-CAU, atpE, atpB*
7	Intergenic region
8	*petL, psbE, psbF, psbL, psbJ, petA, cemA, ycf4, psaI, accD, rbcL*
9	*petG, trnW-CCA, trnP-UGG, psaJ, rpl33, rps18, rpl20, rps12_5’exon, clpP*
10	*rps19_*fragment*, rpl22, rps3, rpl16, rpl14, rps8, rpl36, rps11, rpoA, petD, petB, psbH, psbN, psbT, psbB*
11	*ndhF, rpl32, trnL-UAG, ccsA, ndhD, psaC, ndhE, ndhG, ndhI, ndhA, ndhH, rps15, trnN-GUU, trnR-ACG, rrn5, rrn4.5, rrn23, trnA-UGC, trnI-GAU, rrn16, trnV-GAC, rps12_3’exon, ndhB, trnL-CAA, ycf2*
12	*trnI-CAU*
13	*rpl23, rpl2, rps19_*fragment

infA, rps7, rps16and ycf1were not included in the LCBs as they were not present in all of the plastomes of the putranjivoid clade.

IRs are thought to play a role in stabilizing the plastome (Maréchal and Brisson, 2010). This hypothesis is based on the fact that legume and conifer plastomes, which have no IRs, also show more rearrangements than plastomes containing canonical IRs (Palmer and Thompson, 1982; Hirao et al., 2008; Mower and Vickrey, 2018). The putranjivoid clade is another solid example that increased structural variations coincide with the loss of the IRs. However, species in a lineage of *Erodium*, which also have no IRs, still exhibit a conserved overall plastome structure, resembling those of IR-containing species (Blazier et al., 2016). In contrast, many species of *Geranium* and *Pelargonium* (Chumley et al., 2006; Guisinger et al., 2011; Röschenbleck et al., 2016; Weng et al., 2016) and Campanulaceae (Haberle et al., 2008), some of which have canonical though expanded IRs, possess highly rearranged plastomes. These cases suggest that further comparative study is needed to elucidate the function of IRs in stabilizing plastome structure.

An emerging consensus is that the presence of smaller repeats, rather than the loss of the IRs, is a major driver of plastome rearrangements (Mower and Vickrey, 2018). In the putranjivoid clade, we observed an obvious tendency that plastomes with more genomic rearrangements were also richer in repeats of 30 bp or more (**Table 4**). The number of short repeats are the largest in the *Drypetes* plastomes. While the *Balanops* plastomes, which are the most conserved ones have the fewest number of repeats. Furthermore, more rearrangement events also coincide with the presence of longer repeats (**Table 4**). Being the most rearranged, all *Drypetes* plastomes do possess a pair of sIRs with the length of more than 1,000 bp. As the only case that sIR induced gene duplication found in our study, all *Drypetes* species have two copy of two genes, *psbK* and *trnQ-UUG*, due to the ~1.2kb sIR. Typical IRs in plastomes trigger intra-plastomic homologous recombination, which generates two isomeric plastomes in equimolar abundance (Palmer, 1983; Martin et al., 2014). Multiple studies have detected isomeric plastome structures caused by sIR in several conifers and legumes (Tsumura et al., 2000; Wu et al., 2011; Yi et al., 2013; Qu et al., 2017; Wang et al., 2018). We also confirmed the existence of isomers induced by a pair of 271 bp sIRs in *L. maingayi* (**Figure S5**). Based on our findings, we conclude that smaller repeats indeed have played a role in enhancing plastome structural variation in the putranjivoid clade.

**Table 4:** Number of repeats.

Number of Repeats	Species	Repeat Length/Numbers			
		30-60bp	60-100bp	100-500bp	>1000bp
8	*Balanops balansae*	6	2	0	0
4	*Balanops pedicellata*	4	0	0	0
18	*Lophopyxis maingayi*	13	3	2	0
25	*Drypetes chevalieri*	19	3	2	1
23	*Drypetes diopa*	18	3	1	1
25	*Drypetes hainanensis*	18	3	3	1
37	*Drypetes indica*	20	10	6	1
26	*Drypetes lateriflora*	19	4	2	1
18	*Drypetes longifolia*	14	0	3	1
18	*Drypetes similis*	11	3	3	1

## Data Availability Statement

The datasets generated for this study can be found in the GenBank Database, MN504788–MN504797.

## Author Contributions

T-SY, J-JJ, and D-MJ designed the study. T-SY, J-BY, and D-MJ contributed to tissue sample collections, experiments, and sequences. D-MJ, J-JJ, and LG assembled the plastomes. D-MJ and J-JJ conducted the analysis. D-MJ, T-SY, SW, and J-JJ wrote and edited the manuscript; all authors commented on the manuscript.

## Funding

This project was funded by grants from the Strategic Priority Research Program of the Chinese Academy of Sciences (XDB31010000); the Large-scale Scientific Facilities of the Chinese Academy of Sciences (No. 2017-LSF-GBOWS-02); the National Natural Science Foundation of China [key international (regional) cooperative research project No. 31720103903]; and the open research project of “Cross-Cooperative Team” of the Germplasm Bank of Wild Species, Kunming Institute of Botany, Chinese Academy of Sciences.

## Conflict of Interest

The authors declare that the research was conducted in the absence of any commercial or financial relationships that could be construed as a potential conflict of interest.
