# Supplementary material for: The Loss of the Inverted Repeat in the Putranjivoid Clade of Malpighiales
Source: Front Plant Sci. 2020 Jun 26;11:942. doi: 10.3389/fpls.2020.00942 (PMC7332575; doi:10.3389/fpls.2020.00942)
Supplement: Supplementary file 3 [file Image_1.pdf]

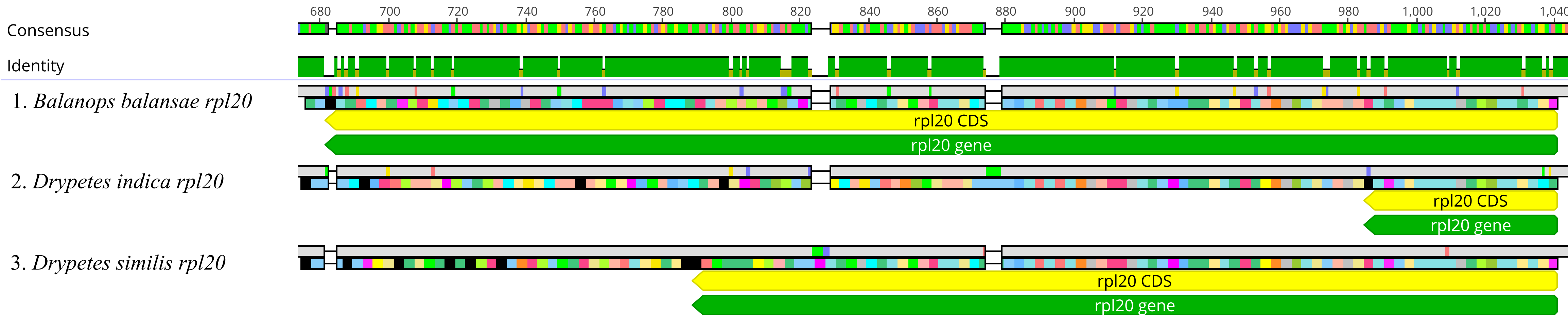


**Figure S1. Alignment of the *rpl20* gene.**

Sequences of *Balanops balansae*, *Drypetes indica* and *D. similis* are included. The black dots represent stop codons.

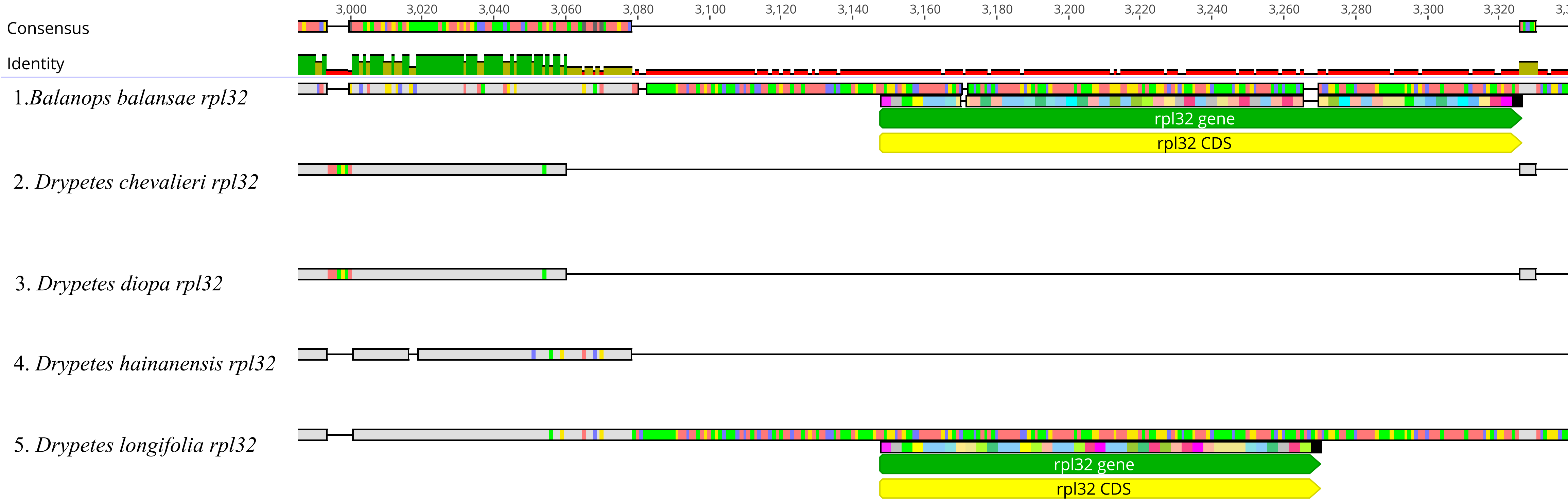


**Figure S2. Alignment of the *rpl32* gene.**
Sequences of *Balanops balansae*, *Drypetes chevalieri*, *D. diopa*, *D. hainanensis*, and *D. longifolia* are included. The black dots represent stop codons.

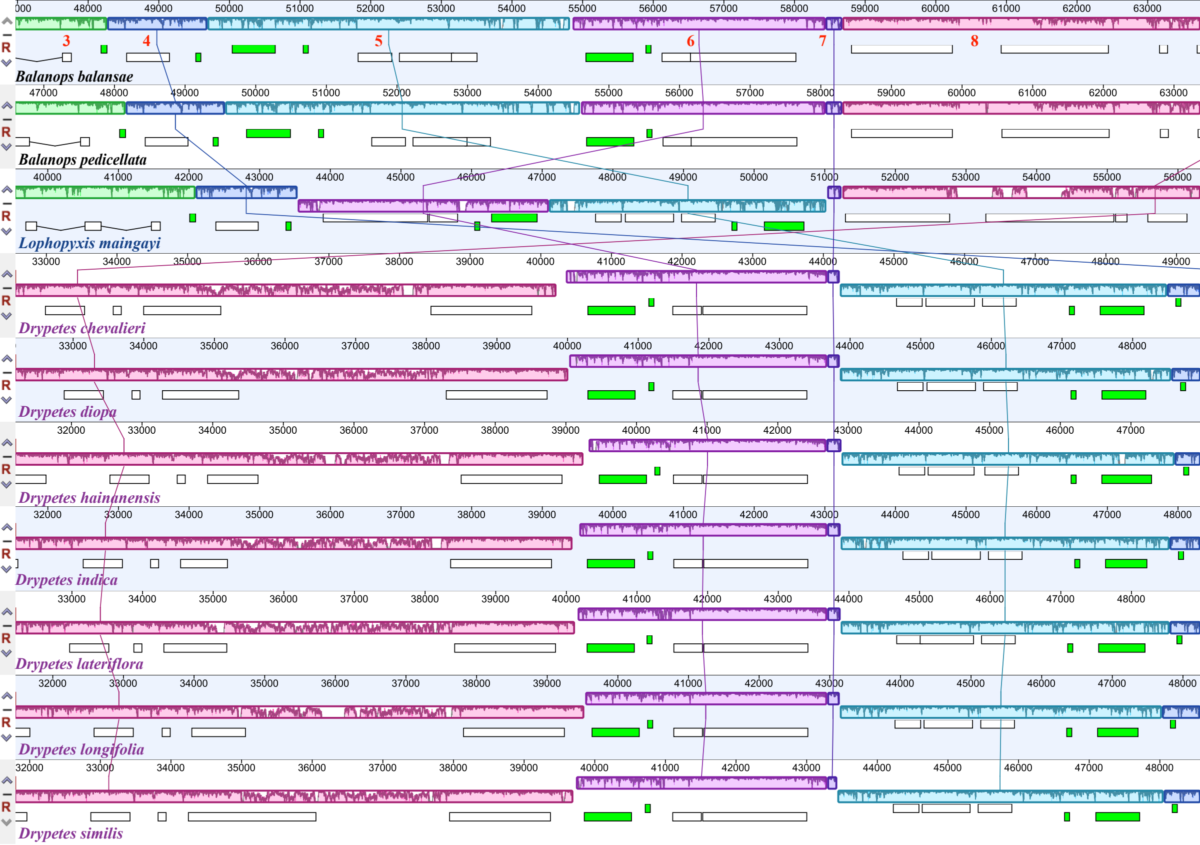


**Figure S3. Close-up for Locally Collinear Block (LCB)7.**

Partial progressiveMauve alignment of 10 Malpighiales plastomes. Color-coded blocks represent syntenic regions. Blocks below the horizontal central line represent inversions relative to the references, shown as the upper two taxa. Species names are color-coded to indicate their family: Balanopaceae (black), Lophopyxidaceae (blue), and Putranjivaceae (purple). The red-colored numbers on the top indicate the corresponding LCBs.

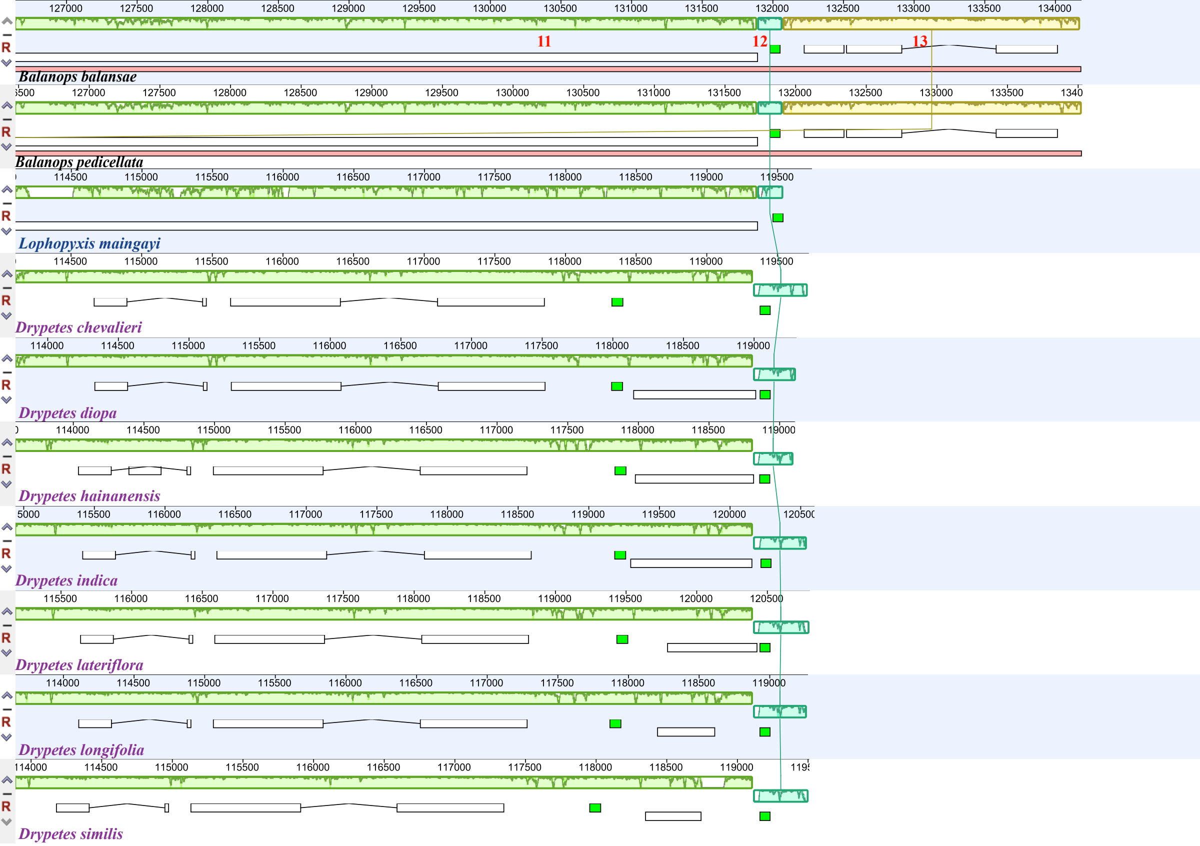


**Figure S4. Close-up for Locally Collinear Block (LCB)12.**
Partial progressiveMauve alignment of 10 Malpighiales plastomes. Color-coded blocks represent syntenic regions. Blocks below the horizontal central line represent inversions relative to the references, shown as the upper two taxa. Species names are color-coded to indicate their family: Balanopaceae (black), Lophopyxidaceae (blue), and Putranjivaceae (purple). The red-colored numbers on the top indicate the corresponding LCBs.

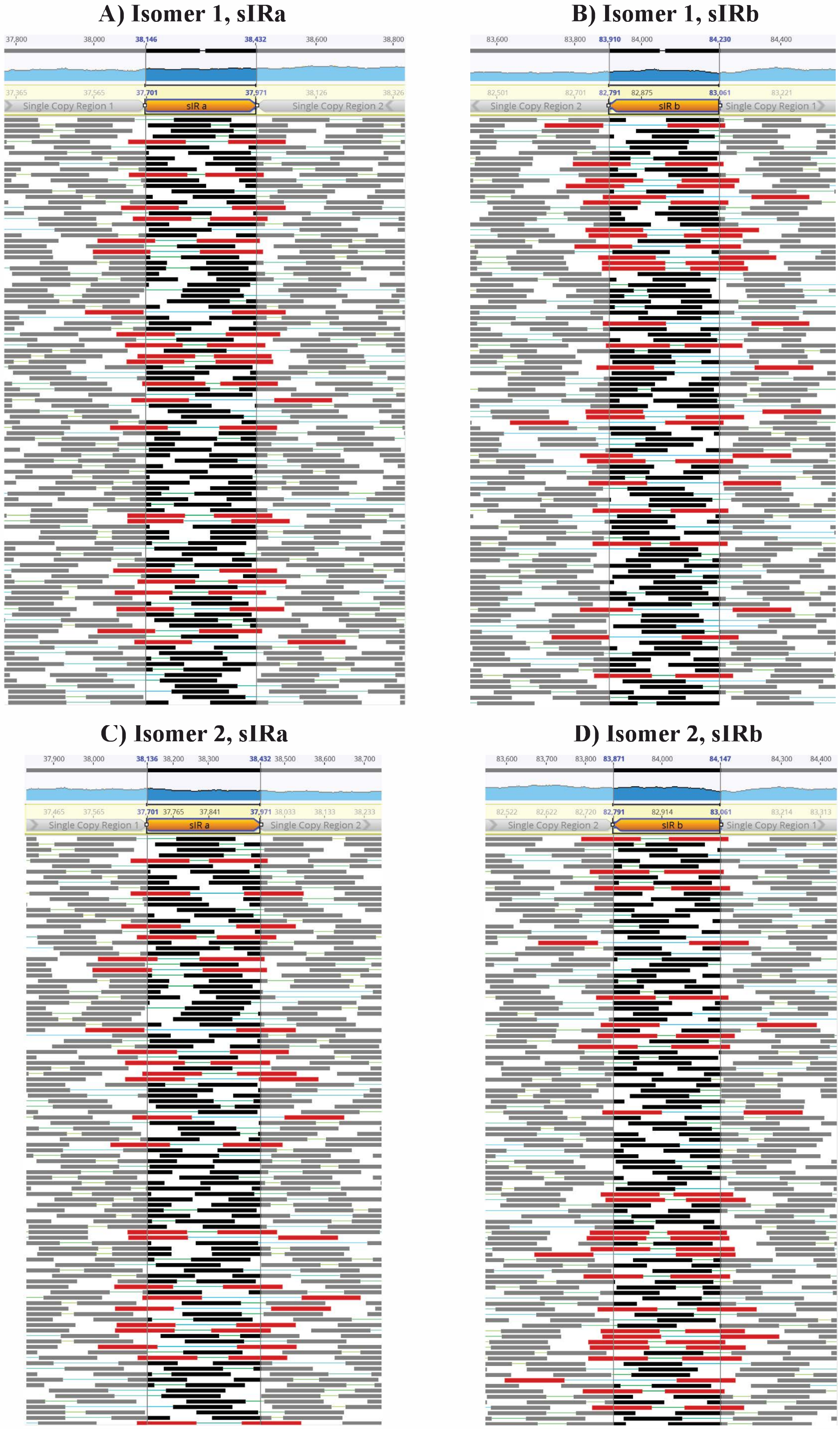


**Figure S5. Partial read mapping results of two candidate isomers of *Lophopyxis maingayi* plastome**

Each candidate isomer is annotated with yellow arrowed bars indicating the sIRs and their directions, with grey arrowed bar indicating the single copy regions and their directions. The black boxes indicate the reads. Each paired reads are linked with a green/blue line. Read pairs spanning the entire sIR are marked with red color. The main difference between isomer 1 (subgraphs **A** & **B**) and isomer 2 (subgraphs **C** & **D**) in this figure is the direction of Single Copy Region 2. Subgraphs **A** & **B** confirmed the existence of isomer 1. Sub-graphs **C** & **D** confirmed the existence of isomer 2.
